# Long Baseline Stereovision for Automatic Detection and Ranging of Moving Objects in the Night Sky

**DOI:** 10.3390/s121012940

**Published:** 2012-09-25

**Authors:** Radu Danescu, Florin Oniga, Vlad Turcu, Octavian Cristea

**Affiliations:** 1 Technical University of Cluj-Napoca, Memorandumului 28, 400114 Cluj-Napoca, Romania; E-Mail: florin.oniga@cs.utcluj.ro; 2 Astronomical Observatory Cluj-Napoca, Astronomical Institute of the Romanian Academy, Cireşilor 19, 400487 Cluj-Napoca, Romania; E-Mail: vladturcu@yahoo.com; 3 BITNET CCSS, Madach 6, 400464 Cluj-Napoca, Romania; E-Mail: octavian.cristea@bitnet.info

**Keywords:** space surveillance, stereo vision, automatic calibration, object recognition

## Abstract

As the number of objects in Earth's atmosphere and in low Earth orbit is continuously increasing; accurate surveillance of these objects has become important. This paper presents a generic, low cost sky surveillance system based on stereovision. Two cameras are placed 37 km apart and synchronized by a GPS-controlled external signal. The intrinsic camera parameters are calibrated before setup in the observation position, the translation vectors are determined from the GPS coordinates and the rotation matrices are continuously estimated using an original automatic calibration methodology based on following known stars. The moving objects in the sky are recognized as line segments in the long exposure images, using an automatic detection and classification algorithm based on image processing. The stereo correspondence is based on the epipolar geometry and is performed automatically using the image detection results. The resulting experimental system is able to automatically detect moving objects such as planes, meteors and Low Earth Orbit satellites, and measure their 3D position in an Earth-bound coordinate system.

## 1. Introduction

Either due to human activity or to Nature, the night sky is filled with moving objects. Planes, meteors, satellites, or even passing asteroids can be observed from Earth, and for many such objects knowing their position is of significant interest. For example, in the context of a permanently increasing number of artificial satellites in the Earth's orbit, continuous monitoring of their position becomes more and more important, so that collisions may be avoided, orbits may be maintained, and de-orbiting events may be accurately predicted. A detailed presentation of the problem of space debris and existing solutions for their observation is found in [1]. In that study we find that RADAR is the preferred technique for observing objects close to the Earth, up to Low Earth Orbit (LEO), but farther away the optical sensors tend to be the best choice, especially when cost effectiveness is taken into consideration.

The easiest and cheapest way to observe the sky is by means of optical systems, cameras and telescopes. The visual data captured by these optical systems can be analyzed through image processing algorithms, in order to automatically detect objects of interest. When two or more optical systems, located in different places on the Earth's surface, are synchronized, and their images are processed together, one can achieve stereovision, a process that relies on the parallax effect to retrieve 3D information of the observed targets directly, without any assumptions on the behavior of the object. Measuring long distances, of hundreds or even thousands of kilometers, requires the use of long distances between the cameras, so that the parallax effect can be significant. We can find many uses of long baseline stereovision, uses that imply accurate long distance measurements, such as cloud height estimation [2] or terrain mapping [3]. Maybe the most impressive deployment of a long baseline stereovision system is NASA's STEREO project, which uses two observation platforms orbiting the Sun at the same distance as the Earth, one station leading and one station trailing our planet, at similar distances. One of the objectives of this project is to study the 3D structure of the Sun's corona, especially the origin of Coronal Mass Ejections. An early presentation of the challenges of this project is found [4]. An overview of the mission is detailed in [5]. Details about the cameras that form the helioscopic imagers, and also about specific challenges regarding calibration and verification of the system are presented in [6].

The most affordable devices for sky observation are off-the-shelf cameras and lenses. These systems can be easily deployed, and what they lack in accuracy is compensated by the ease of setting up and operation. This paper describes a large baseline stereovision system built with low cost components, which can measure with reasonable accuracy the position of objects ranging from 100 to 1,500 km above the ground surface, a range which includes meteors and LEO satellites. This range is conditioned by the baseline, which does not permit a smaller distance, as the large disparity prevents objects such as planes to be observed simultaneously in both images (but they are still detected in a single image), and by the sensitivity of the CCD off-the-shelf camera which does not permit far, faint objects to be observed. Both these problems could easily be overcome with a different geographical setup and a different choice of imaging hardware.

In the first step of our proposed method, the moving objects are detected in each digital image, based on their unique appearance. A defining characteristic of these objects is the linear nature of their image trace, which discriminates them from the point-like stars. The line segment trajectory is caused by the speed of the object, combined with the high exposure time of the imaging device. The night sky images contain a multitude of objects like stars, clouds, condensation trails, outliers that might make it harder to observe the relevant objects' streaks, as well as to distinguish between different types of objects. To perform these tasks accurately, a proper approach would be to detect and classify all the above mentioned objects into their corresponding classes.

A related approach for detection of satellites' streaks in astronomical images is found in [7]. First, image-plane artifacts and large scale background objects are removed. A noise threshold is determined for each image pixel, and objects above this threshold are identified. The detected objects are finally classified as point-like or streak-like, based on image moments. Another relevant approach for moving object detection as image streaks is presented in [8]. The approach is based on a series of steps, the most important ones being background light estimation and removal, star detection and removal, followed by an iterative matched filter used to detect streaks.

Our approach for image streak detection is based on background estimation and removal from continuous image sequences. The features that do not conform to the background are labeled and classified, and the image objects that look line segments are selected for further processing. The stereo matching is performed using the epipolar constraint, which greatly reduces the search space, and which also provides a fast and robust solution for the sub-pixel correspondence. The enforcement of the epipolar constraint and the triangulation process that leads to the 3D coordinates of the detected objects are possible due to an original solution of automatic re-calibration of the extrinsic camera parameters.

This paper will highlight the following contributions:

- A cost effective solution for large baseline stereo image acquisition,
- An original method for automatic calibration of the rotation matrices, based on tracking known star positions in the image sequence,
- An original method for detection and classification of the moving objects in the image sequence,
- An original, computationally efficient method for stereo matching based on the epipolar geometry and the linear trajectory of the moving object.

## 2. Setting up the Large Baseline Stereoscope

### 2.1. The Stereo Image Acquisition System

**Figure 1.**
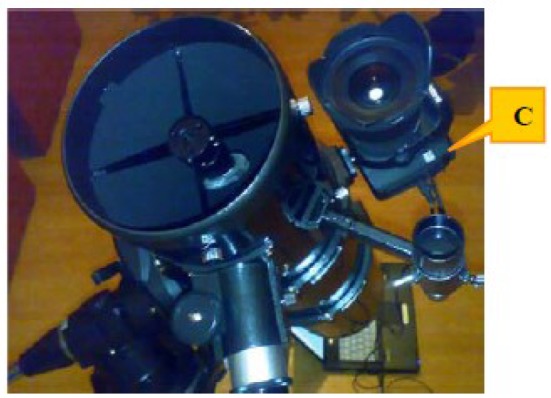
Detail of the individual observing camera. The camera *C* was piggybacked on the telescope, fixed on a tracking-capable equatorial mount Celestron CG5. The telescope itself is not used.

The large baseline stereovision system is composed of two identical observation stations, each station having the following components (see Figure 1):

- “Wide-angle” objective with a focal distance of 20 mm, sigma EX 20 mm F1.8 DG RF aspherical type [9].
- CCD camera, DSLR type (Canon EOS 50D) with a CCD CMOS APS-C chip (22.3 mm × 14.9 mm, 4,572 × 3,168 pixels, in color) [10].
- Equatorial tracking mount, type Celestron CG5 [11].
- Laptop computer equipped with a custom USB to TTL interface for camera triggering. The triggering is done using the remote cable interface of the camera.
- GPS Time Receiver (20 channels) for time synchronization and also for the measurement of the observer's location.

Each camera captures images of the night sky and sends captured images to a local computer. The stereoscope's base-line (the distance between the camera systems) is 37 km, a compromise between simultaneous detection of low altitude objects from two locations and triangulation accuracy.

The theoretical visual FOV covered by this system configuration has a diagonal of 68 degrees. We have tested different exposure times for maximum sensibility and maximum aperture of the objective (ISO 3200, F1.8), and we found that the optimum exposure time was 5 s. The acquisition cycle is comprised of: the exposure time, the analog/digital conversion time and the transfer/storage time (computer and/or camera memory card). The analog/digital converter afferent to the DSLR camera works on 14 bits. To satisfy the resolution and speed requirements previously mentioned we have worked in binning mode—“sRAW2” (2,376 × 1,584 pixels) or “small JPEG” (2,352 × 1,568 pixels). The acquisition cycle for small resolution mode took a minimum of 7 seconds (5 seconds exposure time), and in the case of direct transfer to the computer it reached 8–9 s. Consequently, this method allowed us to obtain an average of 10 images for a complete passing of a LEO satellite.

For the small JPEG image size, the intrinsic calibration process has computed a focal distance, measured in pixels, of 2,187. For this focal distance, and the baseline of 37 km, the expected stereo measurement accuracy can be computed if we assume the equivalent rectified (canonical) stereoscope having the same parameters. The actual stereo performance, for an epipolar geometry system such as the one described in this paper, depends on multiple factors, such as the orientation of the observed object with respect to the epipolar line, but the canonical equivalent error estimation is still a good predictor. For the canonical case, the distance *Z*, the baseline *b*, the focal distance *f* and the disparity *d* are related as Z = bf/d. For our baseline and our focal length, if we assume plus or minus one pixel of error in disparity estimation, we get error values ranging from 4 km at 400 km of distance (1% error), to 55 km at 1,500 km (3.7%), that is, from less than 1% to 4% for the whole LEO range. The error decreases linearly with the baseline. A double baseline would mean half the error, but it can also mean that we get less overlapping field of view between the cameras. A double focal length would mean a similar halving of the error, but with the cost of decreasing the field of view.

### 2.2. Camera Synchronization

**Figure 2.**
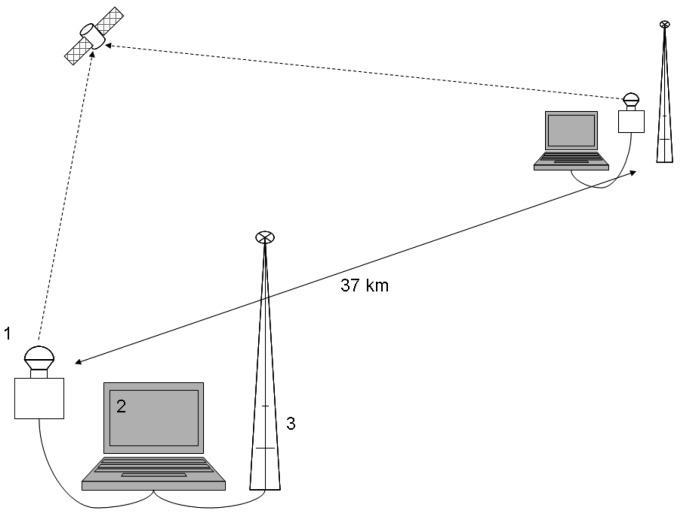
Architecture of the stereoscope. The computer 2 controls through the custom USB to TTL interface the triggering sequence for the camera 1. GPS Time Receiver 3 delivered time signals for synchronization.

Stereovision requires synchronized image acquisition from the two cameras. In a classical stereo system the synchronization is achieved by triggering the cameras by a common signal. However, this solution cannot be applied when the cameras are 37 km apart. Thus, we need two independent units that can generate triggering signals at the same time, without a physical connection between them (see Figure 2). Each unit has a schedule list of triggering times, and if the units have a common schedule, the triggering signals will be simultaneous. Unfortunately, working with a schedule means assuming that the two synchronization units have a precise internal time, which is an assumption that for a common laptop PC is not true. Even if the internal clocks are precisely synchronized, they will quickly fall out of sync.

In the absence of a precise time base, we needed some external signal to keep the two units in sync. This signal is the GPS radio signal, which is received simultaneously, once a second, all over the world. Each unit receives this signal with the help of a GPS receiver, connected to the PC. The following algorithm shows the operation of a unit, based on GPS reception.

**While** (**NotEmpty**
*ScheduleList*)*TriggerTime* = *ScheduleList*.CurrentTime()*GPSTime* = *GPSReceiver*.GetTimeNonBlocking()**If** (*TriggerTime-GPSTime* < 2 s) **While** (GPSTime < TriggerTime)  GPSTime=*GPSReceiver*.GetTimeBlocking() **End While** *Camera*.Trigger() *ScheduleList*.NextTime()**End If****End While**

The unit reads the current triggering time from the *ScheduleList*. The current global time is polled through the method *GPSReceiver*.GetTimeNonBlocking(), which will deliver the most recent timestamp of a GPS reception, without blocking the execution of the program. If the global time is close to the triggering time, the program goes in the blocking mode. The GPS receiver is queried by *GPSReceiver*.GetTimeBlocking(), a function that will exit only when a fresh signal is received. In this way, there will be a minimum delay between the reception of the signal and the triggering of the camera, if the received time is the one matching the schedule list.

### 2.3. The Coordinate Systems

**Figure 3.**
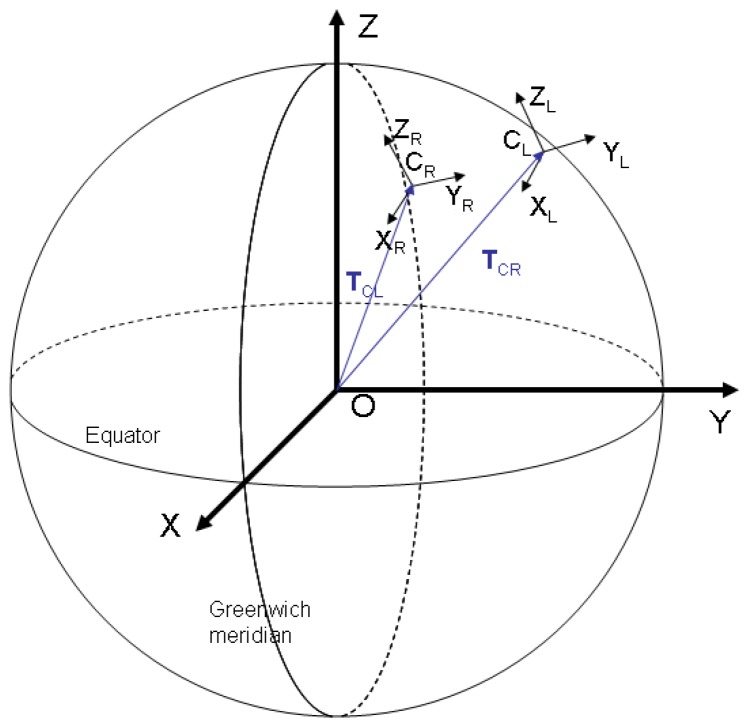
The world and camera coordinate systems.

In order to represent the position of the detected objects, we have chosen a coordinate system whose origin is located in the centre of the Earth, and its coordinate axes are fixed in relation to our planet. Such a coordinate system is the Earth Centered, Earth Fixed (ECEF) one, whose *OZ* axis is the Earth's axis of rotation, pointing to the North Pole, the *OX* axis joins the centre of the Earth with the point on the surface that has zero latitude and longitude, and *OY* is perpendicular to the *XOZ* plane. The equatorial plane is identical to plane *XOY*, and the zero meridian plane is *XOZ* (see Figure 3).

In stereovision terms, we shall refer to the ECEF coordinate system as World Coordinate System, *W_C_*. The two cameras that will observe the sky will have their own coordinate systems, the left camera system centered in *C_L_* and having the axes *X_L_Y_L_Z_L_*, and the right camera system centered in *C_R_*, and having the axes *X_R_Y_R_Z_R_*. The left image plane will be parallel to the plane *X_L_C_L_Y_L_*, and the right image plane will be parallel to *X_R_C_R_Y_R_*.

### 2.4. Camera Parameters

The stereovision process relies on accurate triangulation of corresponding features from the left and right camera images. For this reason, the correspondence between the pixel position and the 3D world must be accurately described by the cameras' parameters. There are two types of parameters, intrinsic and extrinsic.

The extrinsic parameters describe the relation between the coordinate systems of the cameras and the world coordinate system. For each camera, we have a *translation vector*, which describes the position of the camera's optical center in the world coordinate system. We shall denote these vectors by **T_CL_** and **T_CR_**. Conversely, one can express the position of the world coordinate system's origin in the cameras' coordinate systems. These translation vectors are denoted **T_WL_** for the left camera, and **T_WR_** for the right camera.

The orientations of the camera coordinate systems with respect to the world coordinate system are described by the *rotation matrices* of the two cameras, **R_CL_** and **R_CR_**.

Using the translation vectors and the rotation matrices, one can perform coordinate transformations between the world and camera coordinate systems, for any 3D point.

The specific characteristics of a camera plus lens optical system are described by the intrinsic camera parameters (in what follows, we'll denote by the word “camera” the whole camera-lens assembly). For each camera, we'll have the following intrinsic parameters:

- *Focal length*, measured in pixels, which is the distance from the optical centre to the image plane. Each camera has its own focal length: *f_L_* for the left camera, and *f_R_* for the right camera.
- *Position of the principal point*, measured in pixels, represents the intersection of the optical axis of the camera and the image plane. For the left camera, we have ${\text{C}}_{\text{L}}^{\prime }=(x_{\text{CL}}y_{\text{CL}})$, and for the right camera we have ${\text{C}}_{\text{R}}^{\prime }=(x_{\text{CR}}y_{\text{CR}})$.
- The *distortion coefficients* are also included in the intrinsic parameter set. The image distortions are caused by the difference between the ideal pinhole camera model and the real camera and lens assembly. These distortions can be usually modeled by a radial component and a tangential component. The radial distortion of a point in the image, having the coordinates relative to the optical centre *x* and *y*, is the displacement of the point by the following offsets [12]:
  (1) $$\left[\begin{array}{c} \partial x^{r}\\ \partial x^{r} \end{array}\right]=\left[\begin{array}{c} x\cdot \text{(}k_{1}\cdot r^{2}+k_{2}\cdot r^{4}+\ldots )\\ y\cdot (k_{1}\cdot r^{2}+k_{2}\cdot r^{4}+\ldots ) \end{array}\right]$$
  where
  (2) $$r^{2}=x^{2}+y^{2}$$
  and *k_1_, k_2_*, … are the *radial distortion coefficients* (an infinite set, in theory, but in practice one or two are enough to describe the distortion process).

Another type of distortion appears when the curvature centers of the lenses that are assembled in the optical system are not collinear. This distortion has, besides the radial component, a tangential one [13]. The *tangential distortion* is modeled by the following equation:

(3) $$\left[\begin{array}{c} \partial x^{t}\\ \partial x^{t} \end{array}\right]=\left[\begin{array}{c} 2p_{1}\cdot xy+p_{2}(r^{2}+2x^{2})\\ p_{1}(r^{2}+2y^{2})+2p_{2}\cdot xy \end{array}\right]$$

where *p_1_*, *p_2_* are the tangential distortion parameters.

In order to apply the stereovision process, we have to know all the parameters described in this section, for both cameras. The process of computing these parameters is the process of camera calibration, which has two major steps: intrinsic calibration and extrinsic calibration.

### 2.5. Calibration of the Intrinsic Camera Parameters

The intrinsic calibration is performed once, before the cameras are set up in their working positions. The calibration process is a time consuming one, requiring that multiple images containing a chess-like pattern be acquired. The pattern must cover all image regions, and must be photographed from multiple angles and multiple distances from the camera.

The intrinsic calibration is performed using the Bouguet method, available through the Caltech Camera Calibration Toolbox [14]. The method is semi-automatic, relying on the user for selecting the rectangle containing the relevant corners in the image, the individual corners of the chess squares being detected automatically afterwards. The intrinsic calibration process provides the position of the principal point, the focal length, and the distortion coefficients for each camera.

## 3. Online Extrinsic Parameter Calibration

### 3.1. Removal of Distortions

Once the intrinsic calibration process is completed, we know the distortion coefficients that affect the way a scene is represented in the image space. These coefficients cannot be ignored, because a wide angle lens, such as the one used for our night sky observation system, presents considerable distortion towards the image's margins. For this reason, we have to apply a distortion removal algorithm for each image we acquire.

The distortion removal process is applied before extrinsic calibration, and before the process of satellite detection can begin. This way, we can assume that in the next processing steps the images are acquired by an ideal perspective camera (pinhole camera).

The distortion removal algorithm is the following:

For each pixel (*x,y*) from the corrected image **D** (destination):

- Compute the distorted coordinates (*x*′, *y*′) using Equations (1) and (3).
- Assign to the intensity value of image **D** at position (*x,y*) an interpolated value from the distorted source image **S**. We shall use the linear interpolation, which will compute a weighted average between the intensity values located at the neighboring four integer coordinates bounding the real (floating point) position (*x*′, *y*′).

In the next sections, we'll assume that all the images are corrected for distortions.

### 3.2. Computation of the Extrinsic Parameters

The classical extrinsic calibration methodology relies on the following assumptions:

- The intrinsic parameters are known.
- We have a calibration scene, with objects whose 3D coordinates in a World Coordinate System are known. The objects must be easily identifiable, and therefore they are usually special patterns such as targets.
- For each 3D point of known coordinates in the calibration scene, we know its position in the image space.

The calibration process relies on the projection equations, which map a 3D point in the World Coordinate System with its 2D correspondent in the image space. The unknowns of this relation are the extrinsic parameters of the camera: the translation vector and the rotation matrix. Having enough points, an iterative optimization process can extract the unknown values. The precision of the calibration process depends on the number of calibration points, on the accuracy of the measurement of their 3D coordinates with respect to the World Coordinate System, and on the precision of localization of their image correspondents.

The extrinsic calibration must be performed with the cameras in their final position for stereovision-based measurements, and the size of the calibration scene has to be comparable with the distances that we want to measure. In our case, the cameras will be positioned roughly 37 km apart, pointed towards the sky, while the distances we want to measure are in the range of hundreds of kilometers. In these conditions, finding a good calibration scene for a classical calibration approach is very difficult.

To further complicate the matter, the cameras are fixed on equatorial mounts with star tracking capabilities. This mount simplifies the detection process, by making the stars appear static in the image sequence, but in the same time it complicates the calibration process, as it constantly changes the orientation of the cameras with respect to the World Coordinate System.

In this situation, the only calibration objects that we can rely on are the stars, whose position in the World Coordinate System is predictable. Unfortunately, the stars cannot be considered to be objects of known 3D coordinates *X*, *Y* and *Z*, as they are so far away that for all practical purposes their distance from the World Coordinate System's origin can be considered infinite. The only reliable information about the stars is composed of their angular coordinates, and thus they can be used only for computing the camera orientation angles, not for computing the translation vectors.

However, the translation vectors can be determined from the GPS coordinates of the camera's locations. Even if the commercial GPS devices have a low precision, with errors in the range of meters, this error is not critical for our situation, as the distance between cameras (the baseline) is several orders of magnitude greater.

The GPS coordinates of the two observation locations are the following:
*Feleacu*Latitude:46°42′36.50″NLongitude:23°35′36.74″EElevation:743 m*Marisel*Latitude:46°40′34.362″NLongitude:23°07′8.904″EElevation:1,130 m

In order to extract the translation vectors (in the ECEF coordinate system) from the GPS parameters, we'll use the World Geodetic System standard, in its latest revision, WGS 84 [15]. This standard defines the Earth's surface as an ellipsoid with the major radius (equatorial radius) *a* = 6,378,137 m and a flattening factor *f* = 1/298.257223563. The minor (polar) radius can thus be computed as *b* = *a(*1 − *f)*, being approximately 6,356,752.3142 m.

Knowing the latitude *φ*, the longitude *λ* and the elevation *h*, we can extract the translation vector's components *X*, *Y* and *Z* using the following equations (the latitude and longitude angles are converted to radians):

(4) $$c=\frac{1}{\sqrt{\cos^{2}\varphi +{\left(1-f\right)}^{2}\sin^{2}\varphi }}$$

(5) $$s={\left(1-f\right)}^{2}c$$

(6) r=(ac+h)cosφ

(7) X=rcosλ

(8) Y=rsinλ

(9) Z=(as+h)sinφ

**Figure 4.**
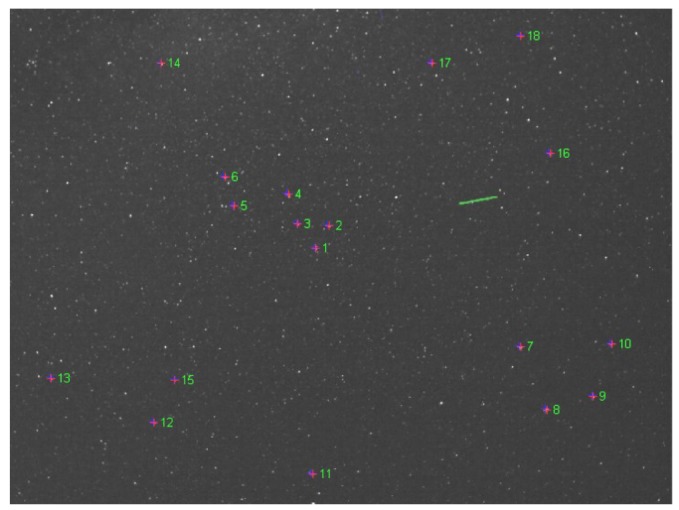
The stars chosen for automatic calibration of the rotation matrices.

At this point, we know the translation vectors **T**_CL_ and **T**_CR_, and the intrinsic parameters of the cameras. All these parameters are fixed, as the camera properties do not change, and the positions of observation remain fixed. The parameters that are still unknown are the rotation matrices **R**_CL_ and **R**_CR_, and these parameters change for each pair of acquired images, as the star tracking system continuously re-orients the cameras. Fortunately, for rotation matrix computation we can use the stars as calibration targets. For a precise calibration, we have chosen 18 stars of high brightness, easily identifiable in the images, which cover as much image surface as possible (see Figure 4).

Table 1 shows the equatorial coordinates [16], for the stars used in calibration process (shown in Figure 4). The Right Ascension (RA) and Declination (DEC) are updated (precession, nutation and proper motion) for the epoch of the observations, 9 July 2011 [17].

As our World Coordinate System is fixed with respect to the Earth, we cannot use, in the process of calibration, the Right Ascension and the Declination directly. We need stellar coordinates that depict the instantaneous positions of the stars, in a specific time instant, with respect to the Earth. The declination angle is related to the equatorial plane, and therefore it remains the same as the Earth rotates, but the Right Ascension is relative to a fixed point in space, and this point changes its position with respect to the World Coordinate System constantly.

The equivalent of longitude for the celestial sphere is the Hour Angle. This angle is formed by the local meridian plane of a place on Earth with the plane passing through the Earth's axis and the sky object to be observed. The Hour Angle is positive towards the West, and is traditionally expressed in time units. The Hour Angle of a star is computed as the difference between the Local Sidereal Time (*LST*) of the place and the Right Ascension (*RA*) of the star. The local sidereal time can be computed from the local time [16].

(10) HA=LST−RA

**Figure 5.**
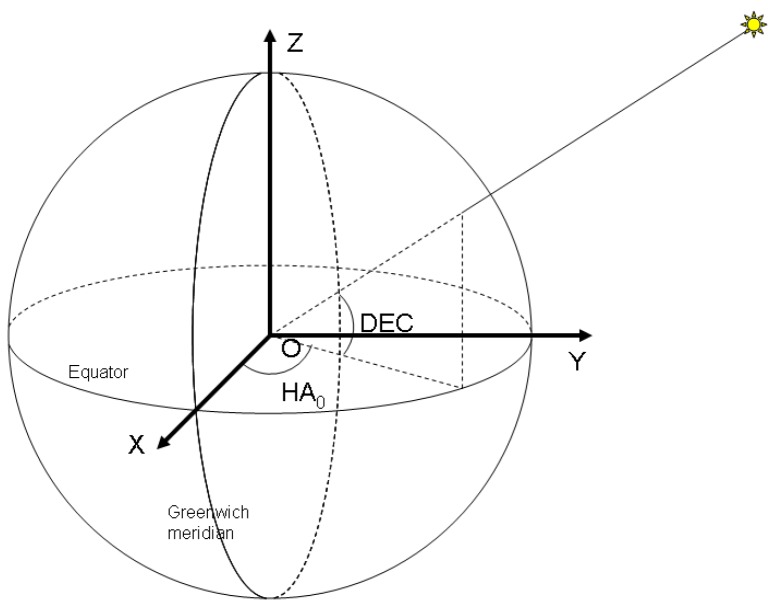
Position of a sky object in Earth-bound polar coordinates.

As the World Coordinate System relates to the Equator and to the Zero Meridian, we find convenient to relate the Hour Angle depicting the instantaneous position of a star to the same Zero Meridian, instead of relating it to the meridian of the place of observation (see Figure 5). In order to find the Hour Angle relative to meridian zero, *HA_0_*, the following steps must be executed:

- Conversion of the local time of frame acquisition to GMT
- Conversion of the GMT time to sidereal time *LST_0_*
- Computation of *HA_0_* as the difference between *LST_0_* and the star's right ascension *RA*.

For a distance R, the polar coordinates can be converted into Cartesian coordinates. The X, Y and Z coordinates of the calibration stars will be computed using the following equations:

(11) $$X=R\cos(\text{DEC})\cos(HA_{0})$$

(12) $$Y=R\cos(\text{DEC})\sin(HA_{0})$$

(13) Z=Rsin(DEC)

At this point we have the 3D position of the stars in the World Coordinate System. In order to apply the rotation matrix calibration, we need to know the position of these stars in the acquired images (after the distortion has been removed from these images).

**Figure 6.**
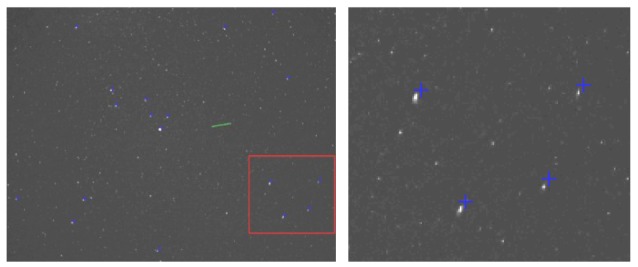
Difference in the manually selected positions of the calibration stars, after a longer period of time since the start of the observation process.

Initially, the position of the reference stars is set manually, for a reference frame for the left (*Feleacu*) and for the right (*Marisel*) observation sources. Ideally, the star tracking system will compensate the Earth's motion and the position of the calibration stars should remain constant for all the image sequence. Unfortunately we have found that small errors may appear and sometimes significant changes are visible, as shown in Figure 6.

As the calibration is very sensitive to the input data, it is crucial that the position of the reference stars is corrected for each frame. The correction process is automatic, using a stereovision-specific correlation technique for finding the correspondent position of the stars across the frames.

**Figure 7.**
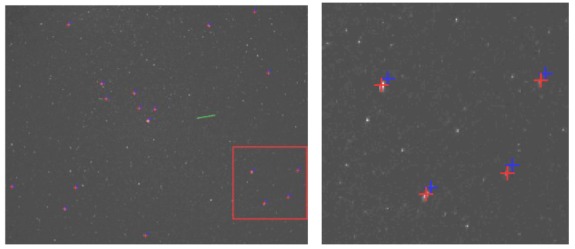
Automatic finding of the new calibration points (marked in red), searching in a neighborhood of the initially marked points (marked in blue).

For each newly acquired frame, we compare image windows in a neighborhood of the reference star position to an original image window of the reference frame, centered in the reference point. A distance between the original window and each of the candidate windows is computed, and the centre of the minimum distance candidate is the new calibration star position. Several window comparison metrics are presented in [18]. The simplest and fastest one is the Sum of Absolute Differences (SAD), applied to a squared neighborhood (5 × 5, 7 × 7, …). For this function, the ideal matching distance is zero, when the corresponding regions are identical. In practice, we'll settle for the minimum in a given search range. The SAD matching function is described by the following equation:

(14) $$\text{SAD}(x_{N},y_{N})=\sum_{i=\frac{w}{2}}^{\frac{w}{2}}\sum_{j=\frac{w}{2}}^{\frac{w}{2}}\left|I_{R}(x_{R}+i,y_{R}+j)-I_{N}(x_{N}+i,y_{N}+j)\right|$$

where (*x_R_*, *y_R_*) is a point in the reference frame, *I_R_* is the reference frame as a matrix of intensity values, (*x_R_*, *y_R_*) is a point in the newly acquired image, *I_N_* is the intensity matrix of the new image, and *w* is the size of the correlation window. For our calibration methodology we have chosen *w* = 11, as the processing time is not critical while the correctness of the correspondence is. The new position is searched in a neighborhood of 41 × 41 pixels around the reference position (see Figure 7).

The automatic adjustment of the calibration points has a success rate of 100% for all frames that we have processed, which means that the calibration of the rotation matrices can be applied automatically, without any supervision, for the whole observation sequence.

The calibration process starts from the equations that project the known 3D reference points to known reference image points. For each point (star) *i* we have a system of two equations:

(15) $$\{\begin{array}{c} \frac{x_{i}-x_{c}}{f}\left[r_{13}\left(X_{i}-X_{cw}\right)+r_{23}(Y_{i}-Y_{cw})+r_{33}(Z_{i}-Z_{cw})\right]+[r_{11}\left(X_{i}-X_{cw}\right)+r_{21}(Y_{i}-Y_{cw})+r_{31}(Z_{i}-Z_{cw})]=0\\ \frac{y_{i}-y_{c}}{f}\left[r_{13}\left(X_{i}-X_{cw}\right)+r_{23}(Y_{i}-Y_{cw})+r_{33}(Z_{i}-Z_{cw})\right]+\left[r_{12}\left(X_{i}-X_{cw}\right)+r_{22}(Y_{i}-Y_{cw})+r_{32}(Z_{i}-Z_{cw})\right]=0 \end{array}$$

where *X_i_*, *Y_i_*, *Z_i_* are the reference point's 3D coordinates, *x_i_* and *y_i_* are the reference point's image coordinates, *x_c_* and *y_c_* are the coordinates of the principal point of the camera, *X_CW_*, *Y_CW_*, *Z_CW_* are the components of the translation vector, and *f* is the camera's focal distance. The unknowns are the rotation matrix **R**'s components, **R** = (*r_kl_*), for *k, l* = 1,2,3.

Due to the fact that the *X_i_*, *Y_i_* and *Z_i_* coordinates of the stars are proportional to their distance from Earth (Equations (11–13)), which is many orders of magnitude higher than the values of the translation vector of the cameras, the coordinates of the cameras in the World Coordinate System *X_CW_*, *Y_CW_* and *Z_CW_* can be removed from Equation (15). Doing this, and using Equations (11–13), we can rewrite Equation (15) such that no assumption about the distance of the stars is needed:

(16) $$\{\begin{array}{c} \frac{x_{i}-x_{c}}{f}\left[r_{13}\cos{\text{DEC}}_{i}\cos{\text{H}}_{0,i}+r_{23}\cos{\text{DEC}}_{i}\sin{\text{HA}}_{0,i}+r_{33}\sin{\text{DEC}}_{i}\right]+\left[r_{11}\cos{\text{DEC}}_{i}\cos{\text{HA}}_{A0,i}+r_{21}\cos{\text{DEC}}_{i}\sin{\text{HA}}_{0,i}+r_{31}\sin{\text{DEC}}_{i}\right]=0\\ \frac{y_{i}-y_{c}}{f}\left[r_{13}\cos{\text{DEC}}_{i}\cos{\text{HA}}_{0,i}+r_{23}\cos{\text{DEC}}_{i}\sin{\text{HA}}_{0,i}+r_{33}\sin{\text{DEC}}_{i}\right]+\left[r_{12}\cos{\text{DEC}}_{i}\cos{\text{HA}}_{0,i}+r_{22}\cos{\text{DEC}}_{i}\sin{\text{HA}}_{0,i}+r_{32}\sin{\text{DEC}}_{i}\right]=0 \end{array}$$

For each star we have two equations, with nine unknowns. The unknowns have only three degrees of freedom, represented by the rotation angles around the coordinate axes, and therefore we can add constraints to model the dependencies between the unknowns. Such constraints can be derived from the fact that the rotation matrix is orthogonal:

(17) $$R\cdot R^{T}=I$$

From this constraint we derive six equations:

(18) $$\{\begin{array}{l} r_{11}\cdot r_{11}+r_{12}\cdot r_{12}+r_{13}\cdot r_{13}-1=0\\ r_{21}\cdot r_{21}+r_{22}\cdot r_{22}+r_{23}\cdot r_{23}-1=0\\ r_{31}\cdot r_{31}+r_{32}\cdot r_{32}+r_{33}\cdot r_{33}-1=0\\ r_{11}\cdot r_{21}+r_{12}\cdot r_{22}+r_{13}\cdot r_{23}=0\\ r_{11}\cdot r_{31}+r_{12}\cdot r_{32}+r_{13}\cdot r_{33}=0\\ r_{21}\cdot r_{31}+r_{22}\cdot r_{32}+r_{23}\cdot r_{23}=0 \end{array}$$

In order to have a number of equations greater or equal to the number of unknowns, we need at least two reference points. For better results, multiple reference points can be used, and for this reason we rely on the known positions of 18 stars. For a set of n ≥ 2 points, we have a linear system of 2n + 6 equations, the first 2n being obtained from system (16) for each point, and the last six equations being the system (18). In order to solve this system, we use the Gauss-Newton iterative method. Starting from an initial guess, which for a rotation matrix can be the identity matrix, gradual corrections are applied until the estimation converges to the right solution [19].

The rotation matrix calibration process is applied independently for both cameras. At the end of this process, the stereoscope is ready to identify and measure the parameters of the moving objects from the image pair.

## 4. Detection of the Moving Objects from Image Sequences

The LEO objects are identified independently in the two images that make up the stereo pair. For each image of the pair we execute the following steps:

- Background removal
- Object candidates detection in the image space
- Object classification

The image pixels that form the possible object are identified as pixels that change in time, with respect to a background. As we use a star tracking system, the only changing pixels that we expect are those caused by a moving object. A simple difference between the frames may be the first solution of choice, but a better choice still is to estimate the background image with a moving average technique, which has a smoothing effect. The value of the averaged background is subtracted from the current frame, and the difference is thresholded using a small enough threshold that the faint contrast objects are still preserved.

**Figure 8.**
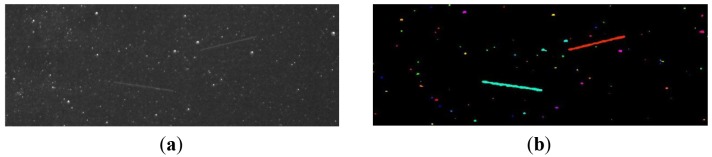
(**a**) The original image containing two satellite streaks. (**b**) Labeled objects on the background-removed image.

Individual image pixel groups are identified by applying a labeling algorithm [20] to the binary image resulted in step 1 (see Figure 8). After that, the labeled binary objects must be classified, so that we can decide whether they are satellite streaks, planes or other objects. In order to perform that classification, we will approximate every binary object by an ellipse, and we'll compute the geometrical properties of this ellipse.

The properties for the image object classification process are: Area, Major Axis Length (*L_MAX_*), Minor Axis Length (*L_MIN_*) and Eccentricity (*e*). These can be computed using the following equations:

(19) $$S_{xx}=\frac{1}{N}\sum_{i=1}^{N}{\left(x_{i}-\bar{x}\right)}^{2}$$

(20) $$S_{yy}=\frac{1}{N}\sum_{i=1}^{N}{\left(y_{i}-\bar{y}\right)}^{2}$$

(21) $$S_{xy}=\frac{1}{N}\sum_{i=1}^{N}\left(x_{i}-\bar{x})(y_{i}-\bar{y}\right)$$

(22) $${L_{\text{MAX}}}^{2}=8(S_{xx}+S_{yy}+C)$$

(23) $${L_{\text{MIN}}}^{2}=8(S_{xx}+S_{yy}-C)$$

(24) $$e=\sqrt{1-\frac{{L_{\text{MIN}}}^{2}}{{L_{\text{MAX}}}^{2}}}$$

(25) $$C=\sqrt{{\left(S_{xx}-S_{yy}\right)}^{2}+{{4S}_{xy}}^{2}}$$

In the above equations, N stands for the number of points of an object (object area in pixels), *x_i_* and *y_i_* are the row and column coordinates of the object's points, and *x̄* and *ȳ* are the coordinates of the center of mass.

In order to build a decision tree, a database for training and testing database was created by manually labeling each object. The result is a database containing all the relevant objects and their classes. Using this database, a decision tree classifier was automatically using Weka [21]:
Area ≤ 85: otherArea > 85| Eccentricity ≤ 0.99| | Eccentricity ≤ 0.935: other| | Eccentricity > 0.935| | | MajorAxisLength ≤ 47.543: other| | | MajorAxisLength > 47.543: satellite| Eccentricity > 0.99| | MajorAxisLength ≤ 200.337: satellite| | MajorAxisLength > 200.337| | | Eccentricity ≤ 0.998: plane| | | Eccentricity > 0.998| | | | MajorAxisLength ≤ 294.676: satellite| | | | MajorAxisLength > 294.676: plane

The decision tree is able to discriminate between three classes of sky objects: satellite (or generic LEO object), plane, and other (which means any image artifact). A more detailed description of the automatic recognition in the image space is presented in [22].

## 5. Computing the 3D Position of the Detected Objects Using Stereovision

### 5.1. Establishing the Correspondence

Stereovision-based 3D reconstruction relies on matching features from the left and right images, followed by triangulation. If the sky objects were point-like in the image space, the correspondence search process would be straightforward. However, we have already seen that this is not the case, and the image signature of these objects is a line segment, having a length proportional to the speed of the object, and to the exposure time of the camera.

If the conditions were ideal, the moving object would become visible at the same time in both images of the stereo pair, and it will also disappear at the same time. If this were the case, the easiest way to find the corresponding points is to look at the ends of the line segments. Unfortunately, there are several causes that make this approach a bad idea. First, the two observation sites have different background illumination conditions, a condition that affects the thresholding of the object's features and may cause different length of segments in the two images. Second, the line segment's length may be influenced by the Gaussian noise of the image, or by the background stars that happen to be near the object, and these conditions may be slightly different for the two cameras. Third, due to the low cost equipment used for camera triggering (off the shelf GPS receivers connected to PC's) there may be some errors in the camera synchronization process, errors that may influence the start of the exposure period (which influences the position of the first end of the linear segment) or the duration of the exposure (which influences the segment length). All these conditions make a compelling argument against using the ends or the middle of the segment for matching. Fortunately, there is a more reliable property of the moving object related line segment, which will accurately allow us to perform accurate, sub-pixel based matching between the images: *the trajectory line itself*.

**Figure 9.**
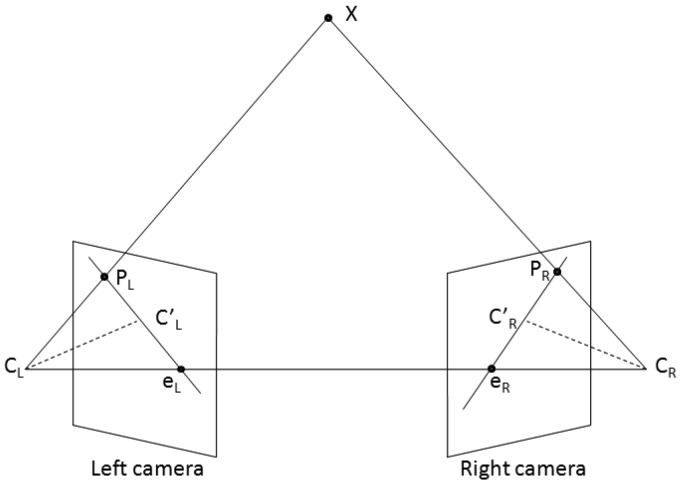
The epipolar geometry.

While the trajectory line is unbounded, and therefore the correspondence points are difficult to find, there is another restriction that drastically limits the search space: the epipolar constraints. The epipolar constraint says that for each point **P_L_** of the left image, the possible correspondences in the right image are located on a line called the epipolar line. This constraint is shown in Figure 9: the optical centers of the two cameras form, together with the unknown 3D point **X** that needs to be measured, a plane that intersects the two image planes forming the epipolar lines. One can see that the plane is completely determined by one projection ray (caused by one point in one of the images) and the line joining the cameras optical centers. This means that knowing a point in the left image and the camera parameters (intrinsic and extrinsic), we can compute the epipolar line that will contain the corresponding right point **P_R_**.

According to [23], the coefficients of the epipolar line *ax* + *by* + *c* = 0 from the right image can be computed using the coordinates of the left image point **P**_L_ = [*x_L_ y_L_* 1] and the 3x3 fundamental matrix **F**:

(26) $${\left[\begin{array}{ccc} a & b & c \end{array}\right]}^{T}={\text{FP}}_{L}$$

The fundamental matrix is computed from the intrinsic and extrinsic parameters of the two cameras [23]:

(27) $$\text{F}={\text{M}}_{R}^{-T}{\text{EM}}_{L}^{-1}$$

where:

(28) $$\begin{array}{cc} {\text{M}}_{L}=\left[\begin{array}{ccc} -f_{L} & 0 & x_{\text{CL}}\\ 0 & -f_{L} & y_{\text{CL}}\\ 0 & 0 & 1 \end{array}\right] & {\text{M}}_{R}=\left[\begin{array}{ccc} -f_{R} & 0 & x_{\text{CR}}\\ 0 & -f & y_{\text{CR}}\\ 0 & 0 & 1 \end{array}\right] \end{array}$$

are the intrinsic matrices of the two cameras, and **E** is the essential matrix, which can be computed as:

(29) E=RS

**R** is the relative rotation matrix between the cameras:

(30) $$\text{R}={\text{R}}_{\text{CR}}^{T}{\text{R}}_{\text{CL}}$$

**S** is determined by the relative translation vector **T**:

(31) $$\text{S}=\left[\begin{array}{ccc} 0 & -\text{T}(3) & \text{T}(2)\\ \text{T}(3) & 0 & -\text{T}(1)\\ -\text{T}(2) & \text{T}(1) & 0 \end{array}\right]$$

The relative translation vector is the difference between the absolute translation vectors of the cameras in the world coordinate system:

(32) $$\text{T}={\text{T}}_{\text{CR}}-{\text{T}}_{\text{CL}}$$

**Figure 10.**
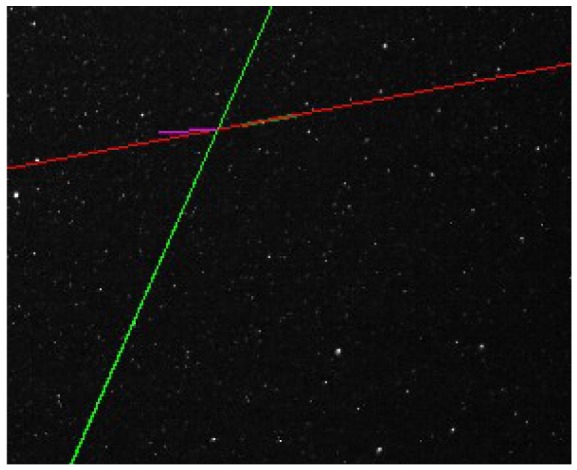
Intersecting the epipolar line (green) and the trajectory line (red) to find the stereo correspondence. The disparity is shown in purple.

As the epipolar lines are easily computed, we can design a simple algorithm for extracting the correspondences between the objects of the left and of the right image. The algorithm will be based on the following steps:

1. Extraction of the center of mass from the objects in the left image. The center of mass will be **P_L_**.
2. Extraction of the axis of elongation (axis of minimum inertia) for the objects detected in the right image. We assume that the moving object will move along this linear trajectory, so that we can safely assume that any error in synchronization will just move the object's position along this line. The axis of elongation passes also through the center of mass of the right image object.
3. Computation of the epipolar line in the right image, by applying Equation (26) to the left image point **P**_L_.
4. Computation of the intersection between the axis of elongation of the right object and the epipolar line caused by the left point. The right correspondent of **P_L_** is constrained to be found on the epipolar line, but it is also constrained to be located on the trajectory of the moving object. Thus, by intersecting the epipolar line computed in step 3 with the trajectory computed in step 2, we find the correspondent point that we are looking for **P_R_** (see Figure 10).

### 5.2. Computation of the 3D Coordinates

The corresponding points in the left and right images are now available, and thus the triangulation process can be applied. This triangulation will transform the image point pair (**P**_L_, **P**_R_) into a single 3D point **P**_W_. This 3D point lies at the intersection of the projection rays passing through **P**_L_ and **P**_R_ (the lines passing through the image points and the optical centers).

Because the camera parameters are known, we can write the equation of the line passing through the 3D point **P**_W_ and the optical center of the left camera **C**_L_ [24]. The perspective projection leads to the following relation:

(33) $$\left[\begin{array}{c} X_{W}\\ X_{W}\\ X_{W} \end{array}\right]=\mu R_{L}\left[\begin{array}{c} x_{L}-x_{\text{CL}}\\ y_{L}-y_{\text{CL}}\\ -f_{L} \end{array}\right]+\left[\begin{array}{c} X_{\text{CL}}\\ Y_{\text{CL}}\\ Z_{\text{CL}} \end{array}\right]$$

where *μ* is a scaling factor depending on the distance *Z_W_*. Multiplying with ${\text{R}}_{L}^{T}$ we obtain:

(34) $$\left[\begin{array}{c} x_{L}-x_{\text{CL}}\\ y_{L}-y_{\text{CL}}\\ -f_{L} \end{array}\right]=\mu^{-1}R_{L}^{T}\left[\begin{array}{c} X_{W}-X_{\text{CL}}\\ Y_{W}-Y_{\text{CL}}\\ Z_{W}-Z_{\text{CL}} \end{array}\right]$$

which can be detailed as:

(35) $$\left[\begin{array}{c} x_{L}-x_{\text{CL}}\\ y_{L}-y_{\text{CL}}\\ -f_{L} \end{array}\right]=\mu^{-1}\left[\begin{array}{ccc} r_{11} & r_{21} & r_{31}\\ r_{12} & r_{22} & r_{32}\\ r_{13} & r_{23} & r_{33} \end{array}\right]\;\left[\begin{array}{c} X_{W}-X_{\text{CL}}\\ Y_{W}-Y_{\text{CL}}\\ Z_{W}-Z_{\text{CL}} \end{array}\right]$$

The above equation is a system of three equations with four unknowns. The third equation can be used to write *μ*^−1^ as a function of the other unknowns:

(36) $$\mu^{-1}=\frac{-f_{L}}{r_{13}(X_{W}-X_{\text{CL}})+r_{23}(Y_{W}-Y_{\text{CL}})+r_{33}(Z_{W}-Z_{\text{CL}})}$$

Replacing *μ*^−1^ in the first two equations, one obtains the following system:

(37) $$\{\begin{array}{c} x_{L}-x_{\text{CL}}=-f_{L}\frac{r_{11}(X_{W}-X_{\text{CL}})+r_{21}(Y_{W}-Y_{\text{CL}})+r_{31}(Z_{W}-Z_{\text{CL}})}{r_{13}(X_{W}-X_{\text{CL}})+r_{23}(Y_{W}-Y_{\text{CL}})+r_{33}(Z_{W}-Z_{\text{CL}})}\\ y_{L}-y_{\text{CL}}=-f_{L}\frac{r_{12}(X_{W}-X_{\text{CL}})+r_{22}(Y_{W}-Y_{\text{CL}})+r_{32}(Z_{W}-Z_{\text{CL}})}{r_{13}(X_{W}-X_{\text{CL}})+r_{23}(Y_{W}-Y_{\text{CL}})+r_{33}(Z_{W}-Z_{\text{CL}})} \end{array}$$

This system has two equations and three unknowns. Writing the same equations for the right camera system, we have a total of four equations with three unknowns, a supra-determined system that can be solved by a least squares approach. From a geometrical point of view, the least squares solution will find the 3D point that has a minimum distance from the two projection lines, even if small calibration or matching errors will prevent these lines from intersecting [23].

## 6. Tests and Results

The experimental system is able to acquire (quasi)synchronized images from the two observation locations set 37 km apart, working autonomously for multiple hours. The acquired images are then processed offline, using an automatic batch processing tool which automatically computes the extrinsic parameters of the cameras for each observation, automatically detects the objects in the two images, performs automatic matching and 3D computation.

The testing process was aimed at assessing two qualities of the system: the capability of detection and recognition of moving objects in a single image, and the performance of the 3D stereo reconstruction.

**Figure 11.**
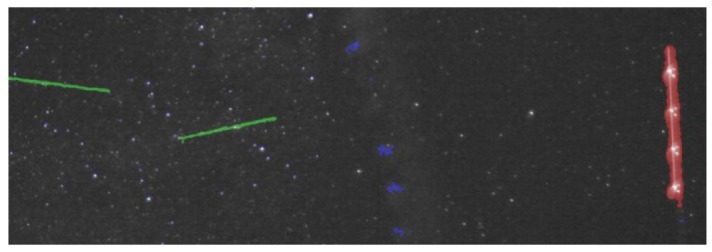
Example of LEO objects classification: green—satellite, blue—outlier (other), red—plane.

**Figure 12.**
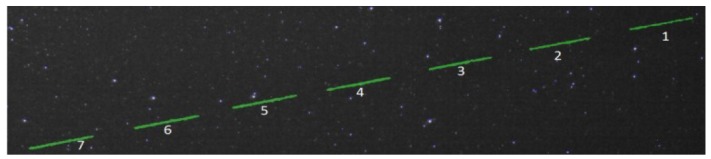
Example of satellite detected in a sequence of images (the detection is superimposed on the first image).

**Figure 13.**
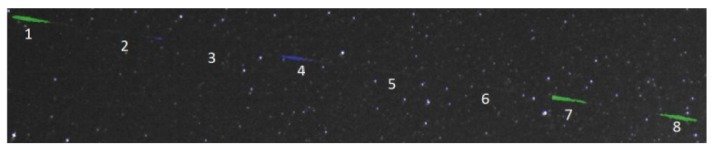
Satellite streaks detection in eight successive frames: very faint or not visible streaks are not detected. This satellite is a spinning one, and therefore its brightness changes as it moves.

For testing the quality of the object classification algorithm, we have used a set of 229 images, each one containing an average of five objects (this includes any blob of image features perceived as moving across frames). The classification results are shown in Table 2, depicted as a confusion matrix:

The weighted ROC area is 0.965, which shows that the classification algorithm delivers robust results. The most important result is that the non-interesting moving features of the image sequence (the “other” class) are never classified as satellites, which means that the false positive rate is near zero. Figures 11–13 show several detection and classification examples.

In order to estimate the accuracy of the 3D reconstruction algorithm, we have focused on LEO satellites, as for some of them the ground truth information can be extracted from the website www.heavens-above.com. We have manually identified several satellites that were visible at the time and place of our observations, and extracted the minimum and maximum distance to the ground of their orbit, as well as the distance to the observation site at the moment of highest elevation.

The primary output of our LEO measurement algorithm is the set of 3D coordinates in the ECEF coordinate system. In order to compute the *distance of the LEO object to the ground*, we compute the distance of the object to the center of the Earth, and subtract from this distance the Earth's radius at the point of observation. The *distance of the LEO object to the observation site* is computed as the Euclidean norm of the difference between the vector containing the 3D coordinates of the LEO and the vector containing the 3D coordinates of the observation site (the translation vector, part of the extrinsic parameters set).

Heavens Above provides the distance to observer information for the LEO object only for the points of appearance, disappearance and maximum elevation. We compare our results to the distance of maximum elevation, but the results may not be entirely accurate as the exact moment of the maximum elevation may not be captured exactly in the image sequence, and the object moves rapidly between frames. Table 3 shows the computed results for several known satellites. The observations were made on 9 July 2011.

Even with the reservations about the accuracy of the ground truth, the results seem to be very promising, both for the distance to observer and for the distance to the ground. With the current limitations of camera sensitivity, the system is able to reliably detect objects that have a distance from the ground in the interval of 100 to 1,500 km.

**Figure 14.**
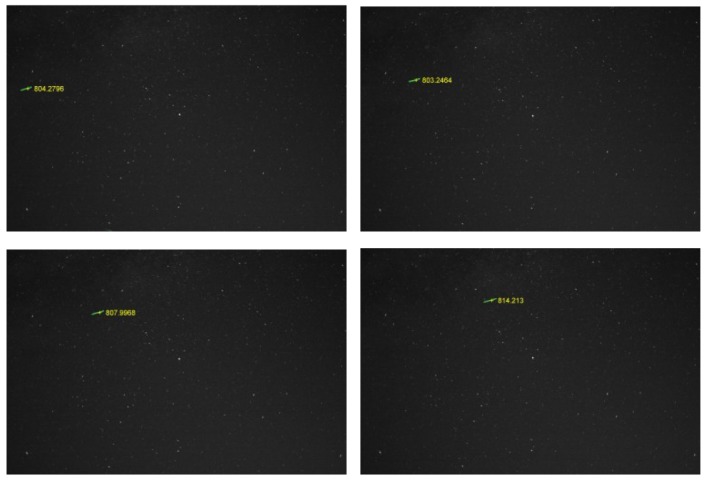

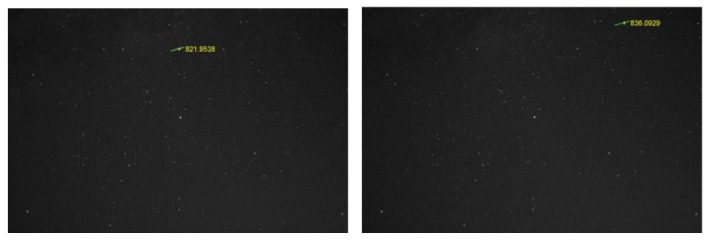
A LEO detected in a sequence of images.

**Figure 15.**
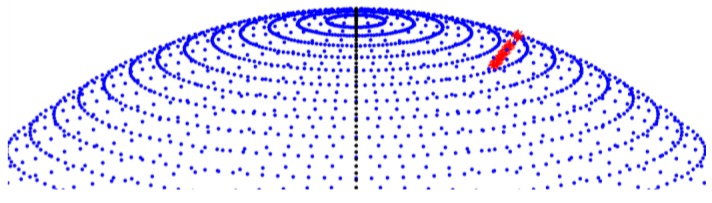
3D visualization of a detected satellite's trajectory with respect to the Earth's surface (MATLAB 3D plot).

Figure 14 shows a sequence of images containing the trajectory of a satellite of height of around 800 km, which is detected and measured in all frames, from the moment it enters the field of view to the moment it exits. Figure 15 shows the 3D position of the satellite with respect to the Earth, clearly showing the satellite's orbiting movement.

## 7. Conclusions and Future Work

In order to detect moving objects in the night sky, several challenges had to be overcome: physical setup of the observation systems, camera synchronization, intrinsic parameter calibration, elimination of the radial distortions, automatic star-based calibration of the extrinsic parameters, automatic detection of object traces in the image space (where the main challenge was the low contrast of the trajectories against the background), automatic computation of the corresponding points from the epipolar lines, and computation of the 3D coordinates by triangulation.

This paper presents a complex, multidisciplinary solution for solving all these challenges, a solution that can be easily implemented with off the shelf components of reasonable cost. The calibration techniques combine classical approaches with the original automatic extraction of the rotation matrix, and the detection and measurement algorithms are original adaptations of computer vision techniques to the particularities of the problem of moving object detection. The resulted solution proved to deliver robust and accurate detection results. The system can be easily extended to run in real time, processing the images as they are acquired, as the computation requirements of the algorithms are light enough so that they fit inside the time between two acquired frames. Setting up a real time system requires broadband communication between the observation sites, which was not available to us, but which is not impossible to set up in other locations.

The functionality of the system can be extended by setting up a database of known satellites that can be automatically identified, combined with a possibility of automatic registration of new satellites as they are detected. Also, the baseline can be reduced in such a way that objects closer to the Earth, such as planes, can also be measured (in our case, the planes are detected, but due to the large baseline they are not seen simultaneously in the two images). The performance of the system can be improved by using cameras of higher sensitivity, or sensitivity beyond the visible spectrum. The visual field can be widened, so that we may cover a larger portion of the sky, or it can be narrowed, through the use of higher focal length optical instruments such as telescopes to increase the detection range so that higher orbit satellites or even near Earth asteroids can be detected. The use of new optical systems will require changes in the intrinsic calibration procedure, as the method currently used will not be suitable anymore.

## Figures and Tables

**Table 1.** Identifiers and equatorial coordinates for the calibration stars.

**Star Number**	**Identifier**	**RA 2011.07.09**			**DEC 2011.07.09**		
		**h**	**m**	**s**	**deg**	**min**	**s**
1	α Lyr	18	37	22.166	38	47	47.569
2	ε_2_ Lyr	18	44	48.144	39	37	36.535
3	ζ_1_ Lyr	18	45	12.593	37	37	9.258
4	δ_2_ Lyr	18	54	56.886	36	54	54.924
5	β Lyr	18	50	32.706	33	22	41.244
6	γ Lyr	18	59	24.873	32	42	26.633
7	γ Dra	17	56	54.988	51	29	23.668
8	β Dra	17	30	44.09	52	17	43.678
9	ν_2_ Dra	17	32	32.237	55	10	3.811
10	ξ Dra	17	53	46.401	56	52	23.452
11	ρ Her	17	24	7.053	37	8	16.503
12	μ Her	17	46	56.876	27	42	52.518
13	SAO 85647	18	2	2.017	21	35	52.639
14	β_1_ Cyg	19	31	13.518	27	59	8.922
15	ξ Her	17	58	15.043	29	14	56.259
16	κ Cyg	19	17	24.883	53	23	28.176
17	δ Cyg	19	45	22.632	45	9	37.064
18	26 Cyg	20	1	43.775	50	8	16.015

**Table 2.** Classification accuracy for LEO hypotheses.

**Classification Result/Ground Truth Class**	**Satellite**	**Other**	**Plane**

Satellite	146	8	1
Other	0	523	0
Plane	2	2	9

**Table 3.** Results of the 3D measurement process.

**Local time**	**Object Name**	**Orbit Min × Max (km)**	**Distance to Observer (km)**	**Distance to Ground, Computed (km)**	**Distance to Observer, Computed (km)**

2:58:51	Cosmos192	704 × 718	714	**704**	**713.70**
3:09:07	Cosmos1743	543 × 564	586	**566**	**582**
3:44:32	Cosmos923Rocket	761 × 789	1078	**790**	**1109**
2:50:59	Cosmos2263Rocket	825 × 848	1161	**806**	**1131**
3:41:44	Meteor *	N.A.	N.A.	**100.25**	**105**

* The detected distance meteor-observer is in the typical range interval for meteor burning in the atmosphere.
